# Lipid Control Post Coronary Artery Bypass Graft: One Year Follow-Up of a Middle-Eastern Cohort

**DOI:** 10.5334/gh.530

**Published:** 2020-02-10

**Authors:** Bassam Atallah, Ramzi Khaddage, Ziad G. Sadik, Saad I. Mallah, Terrence J. Lee-St. John, Shamsah Alfardan, Mahmoud I. Traina, Wael Almahmeed

**Affiliations:** 1 Cleveland Clinic Abu Dhabi, Department of Pharmacy Services, Al Maryah Island, Abu Dhabi, AE; 2 Cleveland Clinic Lerner College of Medicine of Case Western Reserve University, Cleveland, Ohio, US; 3 Cleveland Clinic Abu Dhabi, Department of Family Medicine, Al Maryah Island, Abu Dhabi, AE; 4 Cleveland Clinic Abu Dhabi, Department of Research, Al Maryah Island, Abu Dhabi, AE; 5 Cleveland Clinic Abu Dhabi, Heart and Vascular Institute, Al Maryah Island, Abu Dhabi, AE

**Keywords:** CABG, ASCVD, Hyperlipidemia, Lipid-Lowering-Medications, Statins, Secondary Prevention, Middle-East

## Abstract

**Background:**

Data on patient characteristics and provider practices in the management of lipids per the new guidelines in specific secondary prevention patients in the Middle East is limited.

**Objective:**

To explore patient characteristics and lipid management practices according to the new cholesterol guidelines in secondary prevention patients, up to one year following discharge for coronary artery bypass graft surgery (CABG).

**Methods:**

A retrospective chart review of patients discharged post CABG between February 2017 and February 2018 at a quaternary care centre in the Middle East. Patients were characterized by baseline demographics, comorbidities, and use of lipid lowering medications.

**Results:**

189 patients were included in the analysis. Most were diabetic (70.9%) and classified as very high risk per the ACC/AHA guidelines (84.1%) and as extremely high risk per the AACE guidelines (85.2%). Most patients (93.1%) were discharged on high intensity statin. About one third (28.6%) were never seen or only followed once within the first 2 weeks post discharge. Of those who continued to follow up beyond 3 months and within 1 year of discharge (44.4%), about half (51.2%) had follow-up lipid panels performed. Patients who followed up and were seen by a cardiologist were five times more likely to have lipid panels ordered than those seen solely by a CT surgeon. Of those with follow-up lipid panels beyond 3 months: 59.3% achieved LDL goal of <70 mg/dL and 29% achieved LDL <55 mg/dL based on their respective goals.

**Conclusions:**

Most patients undergoing CABG in a quaternary care centre in the Middle East are high risk ASCVD. Nonetheless, lipid goals are not commonly achieved nor routinely monitored. Providers will need to transition from the previous risk stratification and statin-only focused approach to adopt the most recent guidelines.

## Introduction

Atherosclerotic Cardiovascular Disease (ASCVD) remains the most common cause of mortality and morbidity worldwide [1]. Dyslipidemia in particular is a key factor in the development of atherosclerosis and associated cardiovascular events, with other contributing factors including a family history of heart disease, hypertension, smoking, aging, diabetes mellitus, and other lipoprotein abnormalities [2]. Early risk assessment and intervention have proved to be crucial in the prevention of further events in patients with a history of ASCVD [3].

In patients who have undergone coronary artery bypass graft (CABG) surgery—where severe ischemic coronary disease demands a rerouting of the heart’s blood supply—controlling lipid levels is even more vital than in non-CABG patients. Studies have shown that only 60% of vein grafts remain patent after 10 years from surgery, with 50% of the patent grafts having clinically significant stenosis [4]. Vein graft occlusion rates have also been shown to be 2.1%/year, with vein graft disease appearing at 1 year, and involving 48% of grafts at 5 years and 81% at ≥15 years, with 44% of the latter narrowed >50% [5]. Thus long-term survival and quality of life post-CABG is compromised by the high-risk of developing atherosclerosis in native coronary arteries and bypass grafts, highlighting the importance of lipid control post-CABG.

Lack of attainment of lipid target levels following CABG has been associated with long-term mortality [6]. Various guidelines, such as the 2017 European Academy of Cardio-Thoracic Surgery Guidelines on Perioperative Medication in Adult Cardiac Surgery [7], have been proposed by different international organizations, all of which provide practical guidance for healthcare professionals on the optimal management of ASCVD. However, these guidelines’ specific mention of secondary prevention in CABG patients is very limited. They also differ in their recommendations regarding the appropriate treatment of choice with regard to age, presence or absence of comorbidities, non-statin therapies, and the target goal for LDL-C levels after therapy—which, in the case of secondary prevention (without mention of CABG specifically), the relevant guidelines of the 2013 American College of Cardiology (ACC)/American Heart Association (AHA) decided to shift away from, focusing on a ‘Treat Level of ASCVD Risk’ approach in place of a ‘Treat to Target’ approach [8]. This caused a major shift in practice, with insights from the PINNACLE registry showing that only 20.8% of patients from the participating cardiology practices have had 2 or more lipid assessments after the guideline’s release [9]. Eventually the most recent recommendations re-emphasized the importance of achieving, and thus monitoring for, target levels of LDL-C.

A new Extreme Risk group was introduced in 2017 by the American Association of Clinical Endocrinologists (AACE) for certain secondary prevention patients who would benefit from very low LDL-C targets. The group included anyone with progressive ASCVD, including unstable angina, even after achieving an LDL-C below 70 mg/dL, in addition to established clinical cardiovascular disease in patients with diabetes mellitus, stage III/IV chronic kidney disease, or heterozygous familial hypercholesterolemia. Patients with a history of premature ASCVD were also included in the Extreme Risk group. Treatment goals included an LDL-C <55 mg/dL, with consideration also given to non-HDL-C and Apo B levels [10].

In 2018, new recommendations were introduced by the ACC/AHA emphasizing the LDL goal of less than 70 mg/dL in clinical ASCVD patients and emphasizing the role of non-statin therapies, such as ezetimibe and PCSK9 inhibitors, based on risk assessment and repeat lipid measurements [11]. Statins have been the most commonly prescribed lipid lowering agents, making them the cornerstone of therapy for patients with clinical ASCVD [12]. In fact, the role of statins in acute coronary syndromes (ACS) likely extends beyond lipid lowering to include pleiotropic effects, such as anti-inflammatory and antioxidant properties [13]. However, the more recently approved non-statin Ezetimibe and PCSK9 inhibitors have shown to be a promising addition to the lipid-lowering therapies [14], breaking-ground by their recent endorsement and recommendation in the ACC/AHA cholesterol management guidelines. The availability of new non-statin medications with additional significant LDL reductions, in addition to the most recent recommendations’ re-emphasis on the importance of achieving target LDL-C levels, highlights the importance of following and monitoring for lipid panels in high risk secondary prevention patients to ensure achieving respective goals. New studies have also demonstrated the importance of statin treatment intensification for optimal protection against cardiovascular events in a population with ASCVD. It was shown in the simulation of impact that in a real-world scenario, 818 ASCVD patients in a cohort of 1000 would require treatment intensification with lipid-lowering therapies, resulting in 29 events avoided over 5 years [11,15,16].

Despite these guidelines and evidence-based studies, compliance and optimization of treatment is poor, with a very high percentage of patients still not achieving the recommended lipid targets [16]. ASCVD remains an epidemic both globally and regionally, as the Middle-East continues to have one of the highest cardiovascular disease associated mortalities in the world [17]. In addition, it is known that patients with cardiovascular disease from this region are on average a decade younger than their counterparts in the West and have high prevalence of comorbidities, such as diabetes [18]. There is a lack of data on the appropriateness of lipid control and follow-up per the new guidelines in patients from the Middle East Gulf region following discharge after an atherosclerotic event. This is particularly the case in those who undergo surgical revascularization or CABG, despite their higher risks requiring even greater levels of care.

## Aim of the Study

This study aims to explore lipid management and ASCVD patient characteristics according to the new cholesterol guidelines in secondary prevention patients up to one year following CABG discharge at a quaternary care centre in the Middle East Gulf region. With the lack of data on caregiver practices and patient characteristics in the Middle East, reflecting and constructing solutions to public health epidemics would not be possible. By investigating the current lipid management practice, prevalence of risk, and baseline characteristics of CABG patients, a more holistic understanding of ASCVD in this region can be developed and compared to other demographics.

## Ethics Approval

We obtained Cleveland Clinic Abu Dhabi’s institutional review board approval to conduct this review on patients discharged with a coronary artery bypass graft between February 2017 and February 2018.

## Methods

This single centre retrospective study was conducted in a 364-bedded multi-specialty hospital located in the United Arab Emirates (UAE). We reviewed all patients’ charts discharged with a coronary artery bypass graft between February 2017 and February 2018. Patients were excluded if they were below the age of 18, as well as if they were not discharged alive following revascularization. Data collection was done retrospectively through electronic chart review. Date of CABG and discharge, along with baseline characteristics, including age, gender, nationality, and BMI, were collected and recorded. A past medical history of hyperlipidemia, hypertension, diabetes, and other risk factors associated with ASCVD were also documented. Family history of cardiovascular disease, premature cardiovascular disease, and familial hypercholesterolemia, in addition to the prescribed lipid lowering agents prior to CABG and following discharge were recorded. We classified patients as high risk or extreme risk per the 2017 AACE guidelines and clinical ASCVD or very high risk ASCVD per the 2018 ACC/AHA guidelines. Data collection also included lipid profile results before revascularization and during follow-up clinic visits. Lipid panel results were also documented up to 3 months (visits up to 120 days post discharge were included) and beyond 3 months and up to 1 year following hospital discharge. Baseline characteristics of patients were explored using descriptive statistics.

To examine how physician and patient variables/factors relate to the likelihood of having a lipid panel ordered, a logistic regression model was fit to the subset patients who followed up (N = 84), where the ordering of the lipid panel served as the dichotomous outcome variable. Age (in years), Emirati (vs non-Emirati), BMI, history of diabetes and CVD, high risk classifications as determined by ACCE and ACC/AHA schemes, as well as the treating physician’s specialty, served as the predictor variables. R version 3.5.1 was utilized to perform this analysis.

## Results

A total of 189 patients were included in this study. Table 1 outlines the baseline characteristics and past medical history of our patients as well as their risk classification per the new guidelines. The overall mean age of the cohort was 59.7 ± 10.4 years, with the majority being males (87.8%). Close to half of the patients were UAE nationals (46.6%). The proportion of patients with a past medical history of hyperlipidemia, hypertension, and diabetes mellitus were 90.6%, 82.6% and 71.7%, respectively. Many patients (43.4%) were either current or former smokers. Most patients were being re-vascularized following ACS (67.7%). Of the 189 included patients, 84.1% were classified as very high risk per ACC/AHA guidelines and 85.2% were classified as extremely high risk per AACE guidelines, as shown in Table 1. The very high-risk group included patients with a history of multiple major ASCVD events or one major ASCVD event and two or more ASCVD associated risk factors. Most common risk factors in our study were hyperlipidemia, diabetes, and hypertension. The very high incidence of diabetes contributed to their extreme risk classification as per AACE guidelines. Most patients were already on a statin prior to CABG (91%), and almost all of them were discharged on a statin (99.5%), with the vast majority being a high intensity statin (93.6%). Atorvastatin was the most commonly prescribed therapy, followed by rosuvastatin. Table 2 outlines statin therapy prior to CABG and at discharge.

**Table 1:** Baseline characteristics of patients discharged following CABG (n = 189).

	Mean ± SD/Frequency	Percentage

**Age (years)**	59.7 ± 10.4	N/A
**Nationality – Emirati**	88	46.6%
**Gender – Male**	166	87.8%
Out of Emiratis Only	73	82.95%
**Past Medical History (Most to Least Prevalent)**		
Hyperlipidemia	170	89.9%
Hypertension	157	83.1
Diabetes	134	70.9%
Heart failure	51	27.0%
Prior PCI	47	24.9%
CKD (GFR <60 mL/min)	35	18.5%
PVD	15	7.9%
Uncontrolled Hypothyroidism	10	5.3%
Stroke or TIA	8	4.2%
Prior CABG	2	1.1%
**Body Mass Index (kg/m^2^)**	27.7 ± 4.9	N/A
**Smoker (current or former)**	82	43.4%
**Presentation on Admission**		
ACS	128	67.7%
Stable angina	61	32.3%
**Length of Stay (days)**	8.4 ± 7.5	N/A
**AACE Classification**		
Very high risk	28	14.8%
Extremely high risk	161	85.2%
**ACC/AHA Classification**		
Clinical ASCVD	30	15.9%
Very high risk ASCVD	159	84.1%

**PCI** = Percutaneous Coronary Intervention, **CKD** = Chronic Kidney Disease, **PVD** = Peripheral Vascular Disease, **TIA** = Transient Ischemic Attack, **CABG** = Coronary Artery Bypass Graft, **ACS** = Acute Coronary Syndrome, **AACE** = American Association of Clinical Endocrinologists, **ACC/AHA** = American College of Cardiology/American Heart Association.

**Table 2:** Lipid panel and management of CABG patients (n = 189).

On statins prior to CABG	91.0% (172)

**High intensity**	**72.7%** (125, n = 172)
– Atorvastatin	33.1% (*40 mg*)|18.6% (*80 mg*)
– Rosuvastatin	18.6% (*20 mg*)|3.5% (*40 mg*)
**Moderate intensity**	**24.9%** (47, n = 172)
– Atorvastatin	19.8%
– Rosuvastatin	6.4%
– Other	1.2%
Combination with other agents	7.6% (13, n = 172)
**On statins at discharge**	**99.5% (188)**

**High intensity**	**93.6%** (176, n = 188)
– Atorvastatin	35.6% (*40 mg*)|31.9% (*80 g*)
– Rosuvastatin	18.1% (*20 g*)|8.0% (*40 mg*)
**Moderate intensity**	**6.4%** (12, n = 188)
– Atorvastatin	2.7%
– Rosuvastatin	3.7%
Combination with other agents	5.3% (10, n = 188)
**Availability of lipid panel**	

At baseline	59.8%
Up to the 3 months visit (n = 175)	14.3%
Beyond 3 months visit (n = 84)	51.2%
**Achievement of LDL goal at any follow up beyond 3 months**	

LDL ≤ 100 mg/dL	86%
LDL ≤ 70 mg/dL	58.1%
LDL ≤ 55 mg/dL**	29%

** Percentage out of those deemed extremely high risk per the AACE guidelines (n = 161, 85.2%).

**Figure 1:**
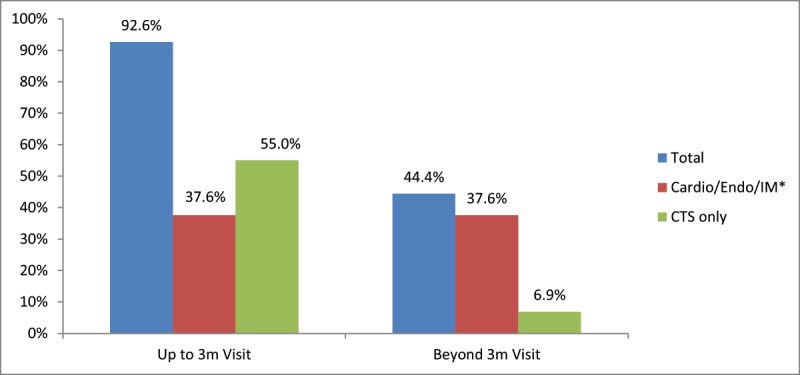
Percentage of CABG patients who completed a clinic visit at the different time intervals (up to 3 months and beyond 3 months) following surgery based on the specialty of the providers. * Patients seen by a medical specialty physician (cardiology, endocrinology and/or internal medicine) might also have been seen by cardiac surgery (CTS).

**Figure 2:**
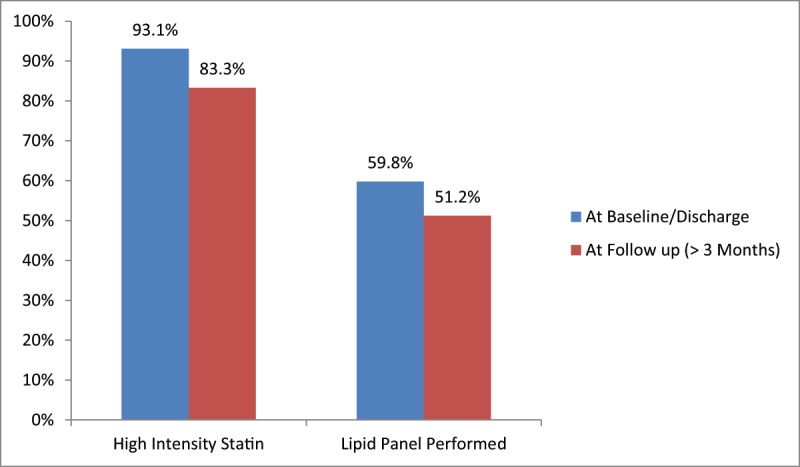
Percentage of lipid panels performed and patients on high intensity statins at discharge and at follow up (beyond the first 3 months).

**Figure 3:**
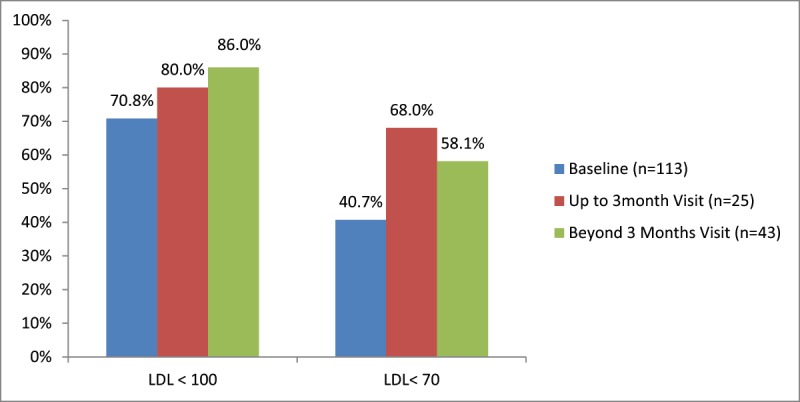
Percentage of patients achieving LDL goals at baseline and follow up visits (LDL in mg/dL).

While most patients (92.6%) completed a clinic visit in the 0–3 month interval, a total of 54 patients (28.6%) were only seen once in the first 2 weeks post discharge or were never seen in clinic. Close to half of the patients (44.4%) continued to follow beyond the first 3 months (Figure 1). Cardiac surgery visits were the most prevalent in the first 3 months; whereas, most patients were also seen by a medical speciality provider (cardiology, endocrinology, internal medicine) beyond that follow-up period. Of those who completed a clinic visit beyond the first 3 months and up to 1 year, 83.3% persisted on a high intensity statin and about half of them had a follow-up lipid panel during this interval (Figure 2). Beyond the first 3 months post discharge, most of our CABG patients (86%) achieved LDL less than 100 mg/dL, many (58.1%) achieved less than 70 mg/dL, but only 29% achieved less than 55 mg/dL out of those deemed extremely high risk per the AACE guidelines (Table 2 and Figure 3).

With regards to patients who followed up, an average of around 6% of interventions that took place were to decrease statin dose, peaking at 13% during the 9–12 months period. After ‘no intervention’ (88.5%), this was the most common occurrence during follow-up on average. In the instances where it was reported, myalgia seemed to be a common reason for a decrease in dosage. An increase in statin dose during follow-up occurred for 3.5% of patients. The addition of non-statin therapies was 0% for PCSK9 and on average 2% for ezetimibe. Table 3 details the interventions performed by quarterly intervals post follow-up.

**Table 3:** Prevalence of different interventions in patients who followed up.

	0–4 Months	5–8 Months	9–12 Months	Beyond 12 Months

**No Intervention**	93.1%	92.3%	81.2%	87.4%
**Statin Dose Increased**	2.3%	2.6%	4.3%	4.6%
**Statin Dose Decreased**	2.3%	3.8%	13.0%	4.6%
**Statin Switched**	0.6%	0.0%	0.0%	1.1%
**Ezetimibe Added**	1.7%	1.3%	1.4%	2.3%
**PCSK9 Added**	0.0%	0.0%	0.0%	0.0%
**Follow Up (n = 189)**	175 (92.6%)	78 (41.3%)	69 (36.5%)	87 (46.0%)

As for the multivariate analysis of the predictors of lipid panel ordering during follow-up, both patient age and physician specialty were found to be significant predictors at the 0.1 level. Specifically, at the 0.05 level, patients seen by a cardiologist had approximately 5 times the odds of receiving a lipid panel when compared to patients seen solely by a Cardiothoracic (CT) surgeon (OR = 4.99, p = 0.045). Additionally, for every year increase in age, the odds of receiving a lipid panel decreased by about 4% (OR = 0.96, p = 0.085). No other predictor was found to be significantly related to the ordering of a lipid panel at the 0.1 level. Table 4 displays the relevant statistics.

**Table 4:** Logistic regression model for predictors of lipid panel ordering in those who followed up beyond 3 months^X^.

Variables	Estimate	Std. Error	Z	p-Value	Odds Ratio	95% CI Odds Ratio	

						min	max

*Intercept*	*2.38*	*2.64*	*0.903*	*0.367*	10.84	0.06	1914.30
Speciality Seen (Cardiologist)	1.61	0.80	2.008	0.045**	4.99	1.04	23.95
Age (Years)	–0.05	0.03	–1.722	0.085*	0.96	0.91	1.01

** Statistically significant at the 95% confidence interval. * Statistically significant at the 90% confidence interval. **^X^** The multivariate analysis included variables of Age, Specialty Seen (CT Surgeon, Cardiologist, Endo), BMI, Nationality, Diabetes, CVD Family History, and Risk Classification per ACC/AHA Guidelines, and AACE Guidelines. Patients who followed up with a cardiologist (p > 0.05) and who were younger (p > 0.1) were more likely to have lipid panels ordered during follow up.

## Discussion

The mean age in this cohort was slightly higher than the GULF RACE-2 registry (59.7 vs. 55 years), which was a prospective registry of Middle Eastern patients with acute coronary events conducted in 6 Gulf countries, with a lower predominance of male gender than in our study (76% vs. 87.8%) [18]. This mean age continues to be around a decade lower than global registries of ACS patients, including the GRACE registry (mean age of 65 years) [20]. In our study, we observed a very high incidence of hyperlipidemia (90.6%), hypertension (82.6%), and diabetes mellitus (71.7%), which are all established to play a major role in the development of ASCVD events [19]. These comorbidities are also remarkably higher than the GULF RACE-2 registry in which 32.7% had hyperlipidemia, 47.2% hypertension, and 39.5% diabetes [18]. This could potentially be explained by our institution being a referral centre in the country in which high risk patients are transferred for surgery.

Compared with lipid registries conducted in the Middle East, our study shows a similarly low achievement of LDL targets (58.1% achieved LDL less than 70 mg/dL and 29% achieved LDL less than 55 mg/dL) in those who had a lipid panel performed following discharge. This is despite the fact that our patients were mostly very high risk secondary prevention patients. In the CEPHEUS study, which was a multi-centre non-interventional survey of patients on lipid lowering drugs in six Arabian Gulf countries including the UAE, LDL target was achieved in only 25% of patients in the highest risk cohort [21]. In the DYSIS registry, a cross-sectional observational multicentre registry of statin-treated outpatients that included the gulf region, 49.5% from UAE and Kuwait met their LDL target, which nonetheless was the highest proportion in that registry [22]. However, they also had one of the highest proportions of “Very high-risk” patients, and it should be noted that LDL-C target levels as of the guidelines used in 2016 did not have targets of <55 mg/dL. In the ICLPS study, a cross-sectional observational study conducted in 18 countries including the gulf region, only 32.1% of the very high-risk patients achieved their LDL goal [23]. However; none of these registries were specifically done in CABG patients but rather included patients on lipid lowering therapies and available lipid measurements. Our study is unique in that it follows lipid control in CABG patients, who are, with no ambiguity, high risk secondary prevention patients and now have designated LDL goals per the new guidelines.

A retrospective study in 2013 looked at CABG patients in an American cohort and found 83.4% of patients to be on lipid-lowering-medication (LLM) at discharge, compared to 99.5% of patients in our study. This difference may be attributed to a growing appreciation of the importance of lowering lipid levels for ASCVD protection. Additionally, the comorbidities present in our population are more prevalent than those found in the study. For instance, in a characteristics comparison between those discharged on LLM and those who weren’t, diabetes seemed to be a significant predictor of LLM use, with an overall diabetes prevalence of 45% compared to 72% in our cohort. LLM use was also associated with an absolute risk reduction of 3.5% of post-discharge death and relative risk reduction of about 80%. Finally, 69.4% of patients (compared to 86% in our cohort) had achieved an LDL value <100 mg/dL, with older age, absent tobacco history, and diabetes again being a significant characteristic in predicting lower LDL achievements in the 2013 study. Additionally, only about 28% of patients achieved LDL levels below 70 mg/dL, compared to 58% in our study. It was reiterated in the discussion of the 2013 US-based study that during that time whether CABG patients should be considered high-risk regardless of cardiovascular risk profile and whether they would benefit from more aggressive LDL reduction <70 mg/dL is not clear [24].

A more recent study looking at attainment of lipid goals post-CABG in an Israeli-cohort found that 44% (vs 58%) of their patients achieved LDL-C levels below 70 mg/dL, with only 43% (vs 72%) having diabetes. The mean age was also higher (65 ± 9 vs 59.7 ± 10.4), with 80% (vs 87.8%) of the patients being male [25]. Targets below 55 mg/dL have not been reported in the studies, owing to its more recent introduction in guidelines.

Because our study was done retrospectively, we were able to examine the naturally occurring physician practices and patient patterns. Nearly all our patients were on lipid lowering agents when discharged and were scheduled for follow-up visits. The fact that most patients (92.6%) completed at least 1 clinic visit within the first 3 months but a majority (56.6%) did not continue to follow up beyond this interval is multifactorial. First, being a referral centre, many of the patients live far from the institution, and while they present for their first follow-up visit following CABG, they continue follow-up closer to home thereafter. Second, underinsured patients accepted for surgery are usually granted only one follow-up visit with the cardiothoracic surgeon after discharge but are not covered for other visits, and thus they also end up following up at other institutions within their coverage network. An important consideration nonetheless is that the patients simply stopped following up or were not requested to do so in the first place—regardless, this would conflict with the recommended guidelines for the care of high-risk patients and may be a result of a perceived lack of need for a lipid target level achievement, and thus monitoring, per the older 2013 guidelines. Lack of LDL target levels may also contribute to not only physician inertia, but also patient passiveness regarding their own care in the absence of a specific, measurable, comprehensible, and tangible goal to work towards and keep them informed of their health.

The 2018 ACC/AHA cholesterol guidelines recommend healthcare professionals assess patients’ adherence and drug effectiveness at 4 to 12 weeks with a fasting or non-fasting lipid profile test [13]. Lipid tests are then re-performed every 3 to 12 months if needed. In our study, we observed that only 51.2% of those patients who continued to follow up beyond the first 3 months after discharge and up to 1 year had a lipid panel done during this time interval. This can be explained by the fact that physicians at that time were still basing decisions on the 2013 ACC/AHA guidelines, which focus on statin intensity only rather than LDL goals. The new 2018 ACC/AHA guidelines now include an LDL goal of less than 70 mg/dL in very high risk ASCVD patients (Class IIa recommendation) and in those aged less than 75 years with ASCVD not at very high-risk (Class IIb recommendation). This is why an LDL goal of less than 100 mg/dL was also looked at, given the fact that lower goals were still given class II grading per the new guidelines, while recognizing that this higher goal is not supported by current guidelines. Physician inertia in failing to order lipid profiles for those who continued to follow up is another contributing factor. This can be particularly relevant because more than half (55%) of the patients who completed a clinic visit within the first 3 months post discharge were seen by cardiothoracic surgeons only with a focus on surgical outcomes rather than medical management. This was confirmed by our multivariate analysis investigating the predictive values for lipid panels being ordered. Patients seen by a cardiologist were up to five times more likely to have lipid panels ordered than patients who followed up with a cardiothoracic surgeon. No data was found on the specific differences in the practices and quality of care with regards to postoperative medical management by cardiologists as opposed to cardiac surgeons, highlighting the need for studies comparing and auditing the practices of both disciplines. One study on a CABG population in the US, however, has previously speculated the existence of clinical inertia with regards to surgeons—it was hypothesized that potential explanations to why some patients were not discharged on LLM included surgeon preference to defer to the patient’s cardiologist [24]. Generally, our data suggests a need for a more multidisciplinary approach to patient care to efficiently and effectively manage from both a medical and surgical perspective. Continuous professional education regarding the importance of lipid target-achievement and the newest guidelines and policies in related but distant fields may also help improve physician practices as a whole. Other important initiatives to improve on this data may also include institutional protocols for management of secondary prevention patients, particularly high-risk CABG patients. Such protocols can be integrated in the electronic health record systems to prompt providers to order baseline lipid panels and implement evidence based dyslipidemia therapy prior to discharge, as well as ordering appropriate follow-up and monitoring.

In the predictive variable analysis, age was also a marginally significant factor (<0.1) in requesting lipid panels. Patients who were older were less likely to have lipid panels ordered, most likely due to a perceived lower life expectancy and thus lower likelihood of a second cardiovascular event occurring. This is not always the case, however, with older patients remaining more at risk for cardiovascular disease than younger patients [2].

During the follow-up period, it was shown that the second most common intervention after ‘none’ was a decrease in statin intensity, most likely due to symptoms of myalgia. Nonetheless, addition of non-statin therapies was 0% for PCSK9 and on average 2% for ezetimibe, despite several studies showing that they can be effective in lowering LDL levels without the common side effects of statins [26]. Thus, an alternative of moderate-intensity statins coupled with non-statin therapies should be taken into consideration to further increase achievement of lipid targets without compromising patient compliance.

Because most patients did not have lipid panels done following discharge and very few had both baseline and follow-up panels, we were not able to fully assess the appropriateness of statin intensification, adding non-statin therapies, or achieving goals per guidelines—and worryingly, neither would have the healthcare professionals managing the patients’ care, highlighting the need for policy changes.

The newly published 2019 guidelines of the European Society of Cardiology (ESC) also classify CABG patients as very high risk, recommending an LDL-C reduction of ≥50% from baseline (defined as the LDL-C level prior to LDL-C lowering medication) and an LDL-C goal <55 mg/dL (Class I recommendation, Level A evidence). Moreover, the ESC suggests that an LDL-C goal <40 mg/dL may be considered for patients with ASCVD who experience a second vascular event within 2 years (not necessarily of the same type as the first event) while taking maximally tolerated statin therapy (Class IIb recommendation, Level B evidence) [27]. Up to one third of the patients included in this study might be candidates for this very low LDL goal given a history of previous stroke, CABG, or percutaneous coronary intervention (PCI). This highlights again the importance of following up on LDL-C levels on all CABG patients by the newest available guidelines.

## Limitations

Our study has several limitations inherent to the retrospective design. First, while our electronic health records system is comprehensive and is integrated with laboratory results and the institution’s outpatient pharmacy, we cannot exclude the possibility that patients were receiving lipid lowering medications from other sources or had lipid panels done at outside facilities. Moreover, although we did not observe readmission for ASCVD-related events in our cohort of patients, we cannot exclude the possibility that some of these patients may have been readmitted at other institutions, and this was not captured in this retrospective review.

Second, many patients did not have baseline lipid panels available and most did not have repeat labs done. Only 54 patients had any lipid panel within 1 year following discharge and out of which only 28 also had a baseline lipid panel. Although this further reflects the gap in practice, and the need for a revision of current policy, it also limits our ability to assess for adherence to guidelines or achieving lipid targets particularly as a percentage reduction from baseline. Nonetheless, an important observation continues to be the fact that a much lower percentage of patients had lipid panels performed than those who successfully completed clinic visits at the different time intervals. For example, while 84 patients completed a clinic visit beyond the first 3 months, only 51.2% of them had a lipid panel done during the same interval. This supports our observation that clinical inertia and statin-focused approaches are noticeable factors contributing to the lack of lipid monitoring.

Third, our centre is a major referral centre in the country and the region for patients with complex cardiovascular conditions requiring high risk procedures. The complexity of patients observed in this study is not necessarily a representation of the average CABG patient in the region. Also, a provider’s decision to order follow-up lipid panels may have been affected by the nature of the patient’s referral for the high-risk surgery and the knowledge that these patients will ultimately continue to follow up medically at facilities closer to home.

## Conclusion

This study retrospectively examined the lipid management of post-CABG patients in a quaternary care centre in the Middle East, a topic and demographic with a paucity of information. Most patients undergoing CABG at a quaternary care centre in the Middle East are at high risk for ASCVD due to the presence of comorbidities. Seeing the lack of lipid monitoring, prescription of non-statin therapies, and recommended LDL level achievements, better lipid monitoring before and following discharge is needed to achieve goals based on the new AACE, ACC/AHA, and the most recent ESC guidelines, accompanied by improved multi-disciplinary teams for effective management of care. Providers will need to transition from the previous risk stratification and statin-only focused approach to adopt the most recent guidelines with consideration of target levels, particularly in high risk patients of the nature of those observed in this study.
